# Individual variation in cognitive style reflects foraging and anti-predator strategies in a small mammal

**DOI:** 10.1038/s41598-019-46582-1

**Published:** 2019-07-12

**Authors:** Valeria Mazza, Jens Jacob, Melanie Dammhahn, Marco Zaccaroni, Jana A. Eccard

**Affiliations:** 1 Animal Ecology, Institute for Biochemistry and Biology, University of Potsdam, Potsdam, Germany; 2 Julius Kühn Institute, Federal Research Centre for Cultivated Plants, Institute for Plant Protection in Horticulture and Forests, Vertebrate Research, Münster, Germany; 3 Department of Biology, University of Florence, Florence, Italy

**Keywords:** Animal behaviour, Behavioural ecology

## Abstract

Balancing foraging gain and predation risk is a fundamental trade-off in the life of animals. Individual strategies to acquire, process, store and use information to solve cognitive tasks are likely to affect speed and flexibility of learning, and ecologically relevant decisions regarding foraging and predation risk. Theory suggests a functional link between individual variation in cognitive style and behaviour (animal personality) via speed-accuracy and risk-reward trade-offs. We tested whether cognitive style and personality affect risk-reward trade-off decisions posed by foraging and predation risk. We exposed 21 bank voles (*Myodes glareolus*) that were bold, fast learning and inflexible and 18 voles that were shy, slow learning and flexible to outdoor enclosures with different risk levels at two food patches. We quantified individual food patch exploitation, foraging and vigilance behaviour. Although both types responded to risk, fast animals increasingly exploited both food patches, gaining access to more food and spending less time searching and exercising vigilance. Slow animals progressively avoided high-risk areas, concentrating foraging effort in the low-risk one, and devoting >50% of visit to vigilance. These patterns indicate that individual differences in cognitive style/personality are reflected in foraging and anti-predator decisions that underlie the individual risk-reward bias.

## Introduction

The ability to gear decisions optimally towards environmental conditions is a fundamental determinant of fitness. Successful reproduction and survival require an efficient estimate of the value of a resource relative to the risk obtaining it. If these assessments are done correctly, the trade-off between gain and safety will not be too expensive^1,2^. Decision-making can be defined as the “process enabling an individual to compare mental representations and choose the most appropriate, given the environmental context”^3^, p.2. Decision-making permeates the every-day life and challenges animals have to cope with, both social and non social, e.g. mate choice, parental care, social behaviour, territoriality, dispersal, foraging, and predator avoidance (e.g.^3,4^). Understanding the determinants and consequences of variation in animal decision-making is therefore a key interest in biology (e.g.^4,5^). Decisions will depend on environmental and social context, physical state, life-history and past experiences (e.g.^5–8^). Also, decisions are often influenced by the individual’s bias in favour of either immediate reward over safety or *vice versa* (e.g.^9–11^). This bias is part of the individual cognitive style/personality make-up^12^.

Personality is defined as the set of individual differences in behaviour that are consistent across time and contexts^13^. Cognitive style refers to the specific strategy by which individuals process, store or act on information to solve a cognitive task (e.g.^12,14,15^), which is expected to be consistent across time and contexts^12,14^. There is growing evidence for the non-independence of animal personality and cognitive traits. Individuals may thus exhibit consistent learning and decision styles (e.g.^12,14,16^). Verbeek *et al*.^16^ hypothesised that such consistent differences could stem from the trade-off between exploration speed and attention to the environment. In a recent theoretical framework Sih and Del Giudice^12^ suggested a functional link between personality types and cognitive styles based on shared risk-reward and speed-accuracy/flexibility trade-offs which is task- and context-dependent. Bolder, more exploratory, proactive individuals are expected to be faster at learning initial discriminations in new activity-based tasks. However, they are also expected to retain a comparatively smaller amount of information for a shorter period of time, form rigid routines and make more errors in tests of avoidance or reversal learning (e.g.^12,14,16,17^). In contrast, shy, slow exploring and reactive animals that are more sensitive to environmental stimuli and more flexible in their behaviour, are suggested to be slower in mastering new activity-based tasks, but also to store more information for longer periods of time and be less challenged in avoidance and reversal learning tasks (e.g.^12,14,16,17^). The two main hypotheses on the evolution and maintenance of syndromes (i.e. consistent sets of correlated traits) suggest that for a syndrome to occur there has to be either an inherent advantage to it, or shared constraints that prevent its breaking up, despite its leading to potentially sub-optimal responses (e.g.^18,19^). If under specific environmental circumstances being bold and fast-learning maximises rewards and being shy and accurate or flexible maximises safety, we should have a co-variation of personality and cognitive style.

Recent studies confirmed the existence of a cognitive style-personality syndrome (e.g.^12^), both in vertebrates and invertebrates (e.g.^20–25^). Most studies addressed the possible link through a speed-accuracy trade-off and reported heterogeneous results regarding the direction of the relationship, both within and between species (e.g.^26^, see also Table 1 in^27^). For example, some studies reported how personality traits can predict either performance accuracy (e.g.^28,29^) or learning speed (e.g.^30–33^). Others reported clear connections between learning performance and behavioural type for specific personality traits (e.g. aggressiveness but not boldness or activity, e.g.^34^, exploratory activity but not aggressiveness or sociability, e.g.^24^). Body condition, sex and environmental conditions were also found to influence the personality/cognitive style link (e.g.^23,35^). Also, recent studies suggest that different personality/cognitive styles might have comparable learning rates but use the learned information quite differently (e.g.^36,37^). Individual differences were also reported in the degree of attention and accuracy devoted to assess a potential threat^38^. Although a general link between risk-taking and learned response to risk was recently found^39^, to our knowledge, Sih and Del Giudice’s^12^ theory on cognitive styles and risk-reward trade-offs received far less attention and was rarely formally and empirically tested (but see e.g.^38^).

At the proximate level, mechanisms that regulate the co-variation of personality and cognitive traits (e.g.^40–44^) show how intraspecific variation in the responsiveness of the hypothalamic-pituitary-adrenal axis to stressors is associated to inter-individual variation in cognitive performance. The pleiotropic effects of hormones such as glucocorticoids can generate co-variation at multiple levels and thus contribute to behavioural and cognitive co-variation^45^. Several behavioural traits that characterize personality and cognitive styles may in fact relate to differences in how individuals respond hormonally to stressors^45^. There is evidence that cognitive performance might be affected by a combination of personality, the kind of reinforcement (positive or negative) used in the learning contingency (e.g.^46^) and the nature of the stress (related or unrelated in time and space with the learning experience, e.g.^47^) experienced by the individual. Also in this case, results seem context- and task-dependent. Specifically, lower levels of corticosterone/cortisol are associated with faster learning (e.g.^41,42^) or higher persistence and number of errors (e.g.^48^). Only a few studies, however, have considered the ultimate consequences of a cognitive style/personality syndrome, which could give insight into its evolution and maintenance (e.g.^39^).

In the present study we investigated whether the same potential constraint acting on both personality and cognitive style (a consistent risk-reward bias) can lead to differences in how individuals face the trade-off posed by foraging and anti-predator decisions. In other words, whether 1) individuals showed a risk-reward bias consistent with the previously assessed cognitive style/personality syndrome and 2) whether this bias was expressed in an ecologically-relevant dimension. Our study species was the bank vole (*Myodes glareolus*), a non-social rodent common in Eurasia that subsists on temporally unpredictable food resources (e.g.^49^) and is subject to intense predation by avian and terrestrial predators (e.g.^50^). Bank voles thus make a suitable study species to address how individuals with different cognitive styles and personality approach the trade-offs caused by the conflicting needs of foraging and avoiding predation.

Based on Sih and Del Giudice’s framework^12^, we predicted that bold individuals with a fast and inflexible cognitive style would preferentially choose immediate rewards at the cost of higher predation risk, while shy individuals with a slow and flexible cognitive style would instead favour safety and experience delayed rewards^12^. We tested these predictions by placing the voles individually in outdoor enclosures provided with different levels of cover and thus presenting zones of different levels of risk. Previous studies on small mammals demonstrated how different cover can be an efficient proxy for exposure to aerial predators and hence be used to assess perceived predation risk (e.g.^51–53^). Bank voles rely only on indirect cues such as cover to infer predation risk by birds, and treat this risk as ubiquitous when cover is low (e.g.^50,54^). This set-up was intended to combine the possibility to closely monitor and quantify individual decision patterns over multiple days with a non-lab setting that would resemble the animal’s natural environment. We monitored the behaviour of the voles while foraging and measured their daily giving-up densities (i.e., the amount of food that foragers leave in a patch composed of a mixture of food and substrate^55^). As the amount of food in a given patch is progressively depleted and the substrate remains constant, the feeding rate decreases and so does the benefit/cost ratio^55^. The giving-up density (GUD) reflects the harvest rate that is not acceptable to justify associated costs and risks^55^. This framework provides a powerful experimental approach with a strong theoretical underpinning to quantitatively measure foraging decisions under varying predation risk (e.g.^55,56^).

## Results

All voles exploited the low-risk area more compared to the high-risk area. Mean GUDs (±S.D.) were 1.08 ± 0.35 g in the low-risk area and 1.4 ± 0.31 g in the high-risk area for fast individuals, and 1.08 ± 0.26 g in the low-risk area and 1.8 ± 0.19 g in the high-risk area for slow individuals. Fast individuals made an average of 4.2 ± 1.9 visits to the low-risk area and 3.5 ± 2.9 visits to the high-risk area, and spent on average 31.4 seconds longer in the low-risk area; slow individuals made an average of 3.2 ± 1.6 visits to the low-risk area and 1.2 ± 1.4 visits to the high-risk area, and spent on average 184.2 seconds longer in the low-risk area.

**Figure 1:**
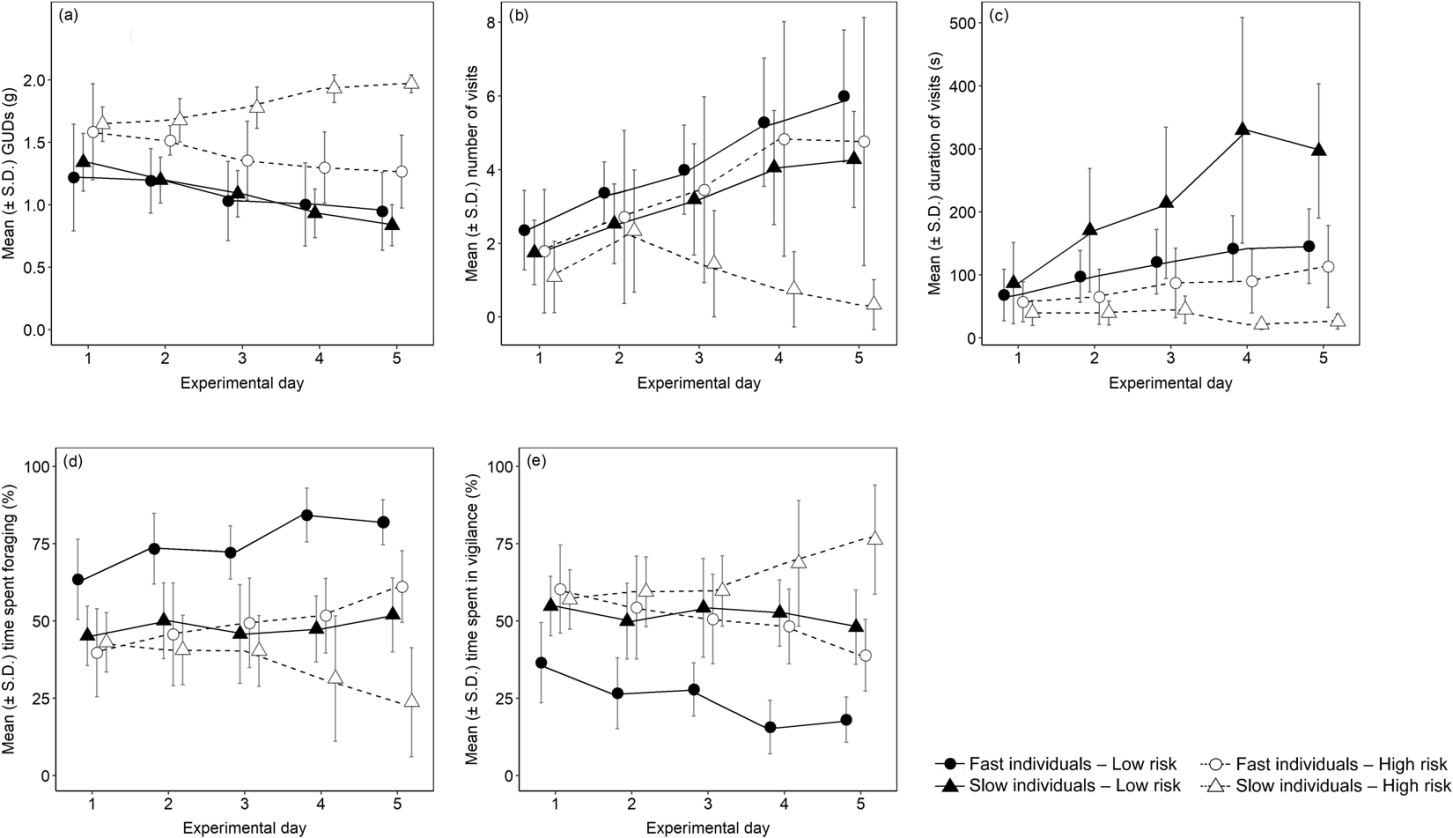
Mean (±S.D.) giving-up densities (**a**), number of visits (**b**), duration of visits (**c**), proportion of time spent foraging (**d**) and in vigilance (**e**) in high-risk and low-risk areas for fast and slow animals for 5 days of observations of 39 individual bank voles (*Myodes glareolus*) in outdoor enclosures. Data points are jittered.

Fast individuals’ GUDs decreased in both risk areas over the experimental days (Fig. 1a, Table 1). The number and duration of visits increased in both areas over the experimental days (Fig. 1b,c, Table 1). Fast individuals spent approx. 75% of their time foraging in the low-risk area (Fig. 1d, Table 1). The amount of vigilance exercised by fast animals was approximately doubled in the high-risk patch compared to the low-risk one (25% in the low-risk area versus 50% in the high-risk area, Fig. 1e, Table 1). The proportion of time spent in foraging increased in both areas over time (Fig. 1d,e, Table 1).

Slow individuals’ GUDs decreased in the low-risk area and increased in the high-risk area over experimental days (Fig. 1a, Table 1). The number and duration of their visits in the low-risk area increased over time, whereas they decreased in number and length in the high-risk area (Fig. 1b,c, Table 1). Slow individuals spent approximately 50% of their visits to the low-risk area in foraging and 50% in exercising vigilance (Fig. 1d,e, Table 1). The proportion of time they spent foraging rather than in vigilance during their visits increased over time in the low-risk area and decreased in the high-risk area (Fig. 1d,e, Table 1).

Fast and slow individuals alike increased the duration of their visits and decreased the proportion of time spent in vigilance at night (Table 1). Fast males made a lower number of visits than fast females (Table 1).

On the last experimental day, all measures of patch use from fast individuals still differed between high- and low-risk patches (GUDs: W = 75.5, P < 0.001; number of visits: W = 1109, P = 0.04; visit duration: W = 1232.5, P < 0.001; foraging: W = 1474, P < 0.001; vigilance: W = 80, P < 0.001).

Food consumption in the high-risk patch was higher in fast individuals (2.99 ± 1.22 g) than in slow ones (1.0 ± 0.48 g) (Mann-Whitney-U test: W = 369, P < 0.001), but did not differ in the low-risk patch (fast individuals: 4.6 g ± 1.37; slow individuals: 4.6 g ± 1.68). Weight loss was slightly higher for fast individuals (−2.43 ± 1.93 g) than for slow individuals (−2.1 ± 1.6 g; Mann-Whitney-U test: W = 3962.5, P = 0.046). Fast individuals had higher body mass at the start of the experiment (fast individuals: 24.7 ± 3.9 g; slow individuals: 23.0 ± 4.3 g; Mann-Whitney-U test: W = 5887.5, P = 0.003). Weight loss in terms of percentage of initial body mass tended to be higher for fast individuals (9.6 ± 8.7%) than for slow individuals (8.7 ± 6.4%; Mann-Whitney-U test: W = 3962.5, P = 0.051)

**Table 1:** Giving-up densities (GUDs), number and duration of visits, proportion of time spent foraging and in vigilance in relation to risk area (high-risk vs low-risk), experimental day, sex and time of day (day vs night) for fast and slow animals for 5 days of observations of 39 individual bank voles (*Myodes glareolus*) in outdoor enclosures.

GUDs	Fast individuals						Slow individuals					
	Estimate	SE	DF		F	P	Estimate	SE	DF		F	P
Intercept	0.65	0.04	1	187	424.4	<**0**.**001**	0.64	0.02	1	159	1127.1	**<0**.**001**
Area (High-risk)	0.19	0.02	1	187	125.4	<**0**.**001**	0.47	0.01	1	159	1017.2	**<0**.**001**
Experimental day	−0.05	0.01	1	187	67.4	<**0**.**001**	−0.07	0.01	1	159	0.4	0.54
Sex (Male)	0.02	0.05	1	19	0.1	0.77	0.003	0.02	1	16	0.02	0.89
Area:Experimental day	/						0.14	0.01	1	159	187.2	<**0**.**001**
**Visit N**	**Estimate**	**SE**		**z**		**P**	**Estimate**	**SE**		**z**		**P**
Intercept	1.39	0.05		25.6		<**0**.**001**	1.13	0.07		16.271		<**0**.**001**
Area (High-risk)	−0.90	0.09		−9.9		<**0**.**001**	−1.68	0.15		−11.063		<**0**.**001**
Experimental day	0.23	0.02		12.6		<**0**.**001**	0.21	0.03		6.958		<**0**.**001**
Sex (Male)	−0.11	0.05		−2.2		**0**.**03**	−0.08	0.07		−1.117		0.26
Time of day (Night)	0.09	0.07		1.4		0.17	0.02	0.08		0.293		0.77
Area:Experimental day	/						−0.48	0.06		−8.119		<**0**.**001**
Area:Time of day	1.10	0.11		0.1		<**0**.**001**	1.06	0.18		5.953		<**0**.**001**
**Visit Duration**	**Estimate**	**SE**	**DF**		**F**	**P**	**Estimate**	**SE**	**DF**		**F**	**P**
Intercept	4.62	0.10	1	367	3944.75	<**0**.**001**	5.08	0.12	1	256	3086.78	<**0**.**001**
Area (High-risk)	−0.77	0.06	1	367	97.52	<**0**.**001**	−2.14	0.10	1	256	706.90	**<0**.**001**
Experimental day	0.20	0.01	1	367	210.62	<**0**.**001**	0.36	0.03	1	256	106.64	**<0**.**001**
Sex (Male)	−0.13	0.14	1	19	0.79	0.38	0.02	0.17	1	16	0.05	0.82
Time of day (Night)	0.09	0.05	1	367	108.87	<**0**.**001**	0.07	0.07	1	256	25.12	<**0**.**001**
Area:Experimental day	/						−0.46	0.05	1	256	95.99	<**0**.**001**
Area:Time of day	0.67	0.08	1	367	75.60	<**0**.**001**	0.68	0.13	1	256	28.73	<**0**.**001**
**Foraging**	**Estimate**	**SE**	**DF**		**F**	**P**	**Estimate**	**SE**	**DF**		**F**	**P**
Intercept	72.96	1.91	1	368	2506.2	<**0**.**001**	46.96	1.44	1	257	3059.7	<**0**.**001**
Area (High-risk)	−25.66	1.12	1	368	513.5	<**0**.**001**	−12.35	1.66	1	257	39.3	<**0**.**001**
Experimental day	4.81	0.40	1	368	146.5	<**0**.**001**	1.09	0.67	1	257	1.1	0.30
Sex (Male)	1.88	2.53	1	19	0.6	0.47	−0.78	1.63	1	16	0.2	0.67
Time of day (Night)	2.37	1.11	1	368	4.5	**0**.**03**	2.97	1.54	1	257	3.8	**0**.**05**
Area:Experimental day	/						−5.36	1.21	1	257	19.7	<**0**.**001**
**Vigilance**	**Estimate**	**SE**	**DF**		**F**	**P**	**Estimate**	**SE**	**DF**		**F**	**P**
Intercept	27.04	1.91	1	368	838.3	<**0**.**001**	53.04	1.44	1	257	4734.8	<**0**.**001**
Area (High-risk)	25.66	1.12	1	368	513.5	<**0**.**001**	12.35	1.66	1	257	39.3	<**0**.**001**
Experimental day	−4.81	0.40	1	368	146.5	<**0**.**001**	−1.09	0.67	1	257	1.1	0.30
Sex (Male)	−1.88	2.53	1	19	0.6	0.47	0.78	1.63	1	16	0.2	0.67
Time of day (Night)	−2.37	1.11	1	368	4.5	**0**.**03**	−2.97	1.54	1	257	3.8	**0**.**05**
Area:Experimental day	/						5.36	1.21	1	257	19.7	<**0**.**001**

Reference levels are given in (). Statistically significant effects are highlighted in bold.

.

## Discussion

Based on GUDs and behavioural observations, we showed that individuals with different cognitive style/personality approach the same risk-reward trade-off in a different way.

According to Sih and Del Giudice’s hypothesis^12^ and our predictions, fast individuals gathered easier to get and greater food rewards at the cost of running higher risks. They increasingly exploited high-risk and low-risk patches, made comparatively short visits, mostly devoted to foraging and not vigilance. Fast voles thus gained access to double the amount of food, reduced search effort and minimised missed opportunity costs.

Fast individuals seemed to trade the ease of foraging for safety, and accept predation risk rather than increasing foraging effort. A riskier strategy is in line with the bolder personality traits fast individuals show (e.g.^12,14,27^). The costs of the risky decisions were probably perceived as less important or more affordable. Individuals that show higher boldness in foraging contexts are also the ones more likely to be bold in predator encounters (e.g.^57,58^). For example, fast great tits (*Parus major*) favoured high-quality food rewards even when the predation risk was experimentally increased^59^. Fast cognitive styles are often connected to inaccuracy and shorter attention spans (e.g.^12^). For example, fast and slow great tits showed differences in attention level even when assessing the risk posed by a potential predator^38^. Fast individuals could have devoted less attention and care in the search for food in the low-risk patch and might have been more prone to switch to the high-risk patch once the low-risk one seemed empty or required extra effort and attention to be completely searched and exploited. Fast individuals are also inflexible and quickly form routines (e.g.^12,14,16,43^). Once they started using both food patches, they might simply have continued to pursue the same course of action (e.g.^60^), facing the inherent risks. The fact that they exploited the high-risk patch significantly less than the low-risk one, however, indicates that they still perceived the differential risk associated with each (e.g.^38,51,53,54^).

Slow individuals minimised predation risk but invested more time and effort in gathering delayed and smaller food rewards. They spent more than half of the time exercising vigilance in both areas, progressively avoided the high-risk area while increasing foraging effort in the low-risk area. This exploitation pattern resulted in diminishing returns, thus increasing missed-opportunity costs. For slow and shy individuals, the best strategy is usually to play it safe and distribute benefits on an extended life-span (e.g.^61–64^). The long time spent in vigilance in the high-risk patch might have prevented efficient foraging (e.g.^65^). Slow, accurate individuals would have been able to conduct a more thorough search for food in the patch that allowed them to meet their needs without resorting to riskier options. Personality and cognitive style both contributed to the decision patterns we observed. If individual decisions were solely driven by risk-propensity or longer habituation rates to a novel environment, shy individuals should have shown a consistent avoidance of the high-risk patch from the start or, possibly, a slow increase in the patch use over the days. Instead these different patterns emerged after a few days in the enclosures, during which all animals behaved in a similar way.

Previous studies, especially in birds, report a higher HPA-activity promotes an increase in activity and locomotion (e.g.^66^), and since slow animals usually display the highest HPA-activity and reactivity in response to stressors (e.g.^67^) it could be argued that the initial pattern of patch exploitation of the slow animals was cofounded by the more pronounced locomotor and foraging activity induced by the stress of being in a novel environment. Studies on mammals, however, report a more complex relationship between activity and HPA-activity and reactivity (e.g.^68–70^) and that rodents can show a decrease both in foraging and locomotor activity following a stressful experience (e.g.^71,72^). In the present study, slow animals responded to the challenge of a novel environment by a decrease in activity as we assessed in the open field^27^. There was also no correlation of activity levels in our experimental animals with measurements of HPA-activity and reactivity (Mazza *et al*., unpublished data). Based on GUDs as well as behavioural data from the videos, we know that slow voles started with some foraging activity in the high-risk patch, so they were visiting it and using it for procuring food. Then the foraging activity decreased while the vigilance increased and the visits to the high-risk patch decreased or stopped. If the visits they made to both trays were caused by heightened locomotor activity induced by fear, we would expect highest vigilance, little or no food consumption during the first visits, and possibly a slow increment of the foraging behaviour when the initial stress of the new environment started to ebb away. Altogether, we believe these patterns point toward an extended exploration, i.e. a prolonged sampling of environmental information that lead to the decision of concentrating the foraging activity on the low-risk patch.

At present we cannot disentangle the effect of personality from the other cognitive components involved in the process of decision-making; we can only report a clear correlation between foraging and anti-predator decisions in outdoor conditions, and the cognitive style/personality syndrome measured in bank voles under laboratory conditions. Designing future experiments that disentangle the two components will be challenging but will shed more light on the mechanisms driving the non-independence of behavioural and cognitive traits.

All voles spent more time foraging and made longer visits to the patches at night. Bank voles are often found to be more active during the night (e.g.^73,74^) and since this pattern did not differ between fast and slow individuals it will not be discussed further.

Unlike^39^ we did not detect sex differences in the observed patterns, with the exception of a lower number of visits for fast males compared to fast females. Bank voles have a polygynandrous, non-resource-based mating system^75^, with sexual dimorphism which might be not pronounced enough to significantly affect their risk-reward biases compared to individual cognitive style and personality. Future studies carried out in a context that emphasises sex differences (e.g. reproductive investment) might illuminate these aspects further.

Fast individuals had probably higher metabolic needs (e.g.^62,76,77^) as shown by higher food intake but changes in percentage of body weight similar to slow individuals’ at the end of the experiment. However, the observed foraging decisions could not be attributed exclusively to physiological demands (e.g.^78,79^). In order to obtain the extra food they might have needed compared to slow individuals, fast individuals were more prone to take risks than put time and effort in intensifying the search in the low-risk patch. These results also seem to indicate that the different foraging strategies adopted by fast and slow individuals yielded similar nutritional advantages. Fast and slow individuals might thus ultimately achieve comparable fitness (e.g.^61–64^). In the last 10 years, theoretical and empirical work (e.g.^62,80^) supported the inclusion of behavioural aspects in the pace-of-life-syndrome (POLS)^81^, a framework for the adaptive integration of behaviour, physiology and life history^82^. So far, this framework has not included cognitive aspects^83^, and empirical evidence supporting a connection between cognitive strategies and physiology is scarce (but see e.g.^83,84^). Future studies clarifying the connections between cognitive and physiological traits, as well as the costs associated to the different strategies might provide support for a more comprehensive view of both POLS and cognitive research.

In conclusion, the results of the present study suggest that the risk-reward trade-off could link personality and cognitive style and might contribute to the evolutionary maintenance of the variation in each domain. More studies are needed to ascertain the consequences of these different strategies on long-term survival and reproductive success. Also, the same decision-making aspects should be investigated under different environmental conditions to assess whether the personality-cognitive style syndrome leads to suboptimal responses or breaks up when different combinations of traits are favoured. To fully illuminate the ecological and evolutionary significance of this syndrome, we need studies of phenotypic (co)variation in syndrome traits that are performed under appropriate ecological contexts of variation in risks and resources, i.e. those where risk-reward trade-offs create variation in fitness outcomes. We offer this as a first step in understanding the mechanisms underlying the maintenance of the variation in decision-making processes and how this variation is connected to individual differences in cognition and behaviour.

## Methods

### Animals and housing

Bank voles (30 males and 30 females) were born in captivity from 25 different litters. The parents were either wild-caught or removed from the wild since 1 to 4 generations and originated from 5 populations in the area around Potsdam (Germany). Animals were housed individually, under ambient light, temperature and humidity, that mirrored the outside conditions, in standard laboratory cages (36 × 21 × 15 cm). The holding room was adjacent to the enclosures and the window was kept open, so noises and odours coming from the enclosures and their surroundings were already experienced by the voles.

Cages were provided with wood shavings and hay as bedding, and a cardboard shelter. Commercial food pellets (Altromin 1324; Altromin Spezialfutter GmbH & Co.KG, Lage, Germany) and water *ad libitum* were supplied at all times. Bedding was changed every week.

Of the 60 individuals, 9 (4 males and 5 females) were used for pilot tests and, thus, were not involved in the main experiment, 7 (4 males and 3 females) died before being tested in the enclosures, and 5 (4 males, 1 female) died during the enclosures trials. These losses were ascribed to natural causes as age of the voles was above the average voles’ life expectancy (14–17 months) and therefore, mortality rates were within the normal range (e.g.^85^). Consequently, we report results for 39 voles: 21 bold, fast learners (10 males and 11 females) and 18 shy slow learners (8 males and 10 females).

### Assessment of cognitive style and personality, and choice of experimental groups

Individuals were assessed for cognitive style and personality. A detailed description of testing procedures is provided in the appendix A in the Supplementary Material and in^27^. Briefly, we tested the voles for their olfactory associative learning speed and flexibility in a reward contingency. We also assessed repeatable among-individual differences in activity, exploration and boldness. The 39 voles involved in the present study were the ones that displayed the fastest and most inflexible cognitive style as well as being consistently bold and active (N = 10 males, N = 11 females, Fig. S1 in the Supplementary Material) and the slowest and most flexible cognitive style as well as being consistently shy and less active (N = 8 males, N = 10 females, Fig. S1 in the Supplementary Material). These individuals are termed fast and slow individuals from here on.

### Enclosure trials

Experiments were conducted in four 3 × 4 m outdoor enclosures under controlled conditions. Enclosures were protected against predators and weather conditions by mesh wire walls and a plastic roof cover 3 m above the enclosures. Enclosures contained perennial grassland vegetation. We divided each enclosure into two areas of 2 × 3 m: a low-risk, high-grass area with ca. 20 cm vegetation height, and a high-risk low-grass area with ca. 2 cm vegetation height. We added camouflage netting (2 × 3 m) (ca. 20 cm above the grass) in the low-risk area to provide additional cover. The position of the two low- and high-risk areas was alternated across enclosures. In each enclosure a plastic nest box (32 × 22 × 16 cm) provided with hay was buried level with the enclosure surface in one corner of the low-risk area to provide a nesting opportunity.

A plastic tray (20 × 15 × 5 cm) was placed level with the enclosure surface in the middle of each low- and high-risk area to create an artificial food patch. Each tray contained 2 g of crushed hazelnuts mixed into 0.75 l of sand^56^. Preliminary trials were run to test the appropriateness of substrate and food type and quantity, as well as the overall structure of the feeding station^86^. The animals used for these trials were only used for piloting and are therefore not included in the experimental dataset (see above “Animals and housing”). At the end of each trial, enclosures were watered and mowed to the desired vegetation height, and the hay in the nest-box was replaced. Each enclosure was occupied by a single vole at a time. The combination of individual’s cognitive style, sex and enclosure was randomized across trials.

Experiments were conducted from June to September 2016. At the start of each trial, each vole was taken from its home cage and weighed with a spring scale (PESOLA AG, Schindellegi, Switzerland) to the nearest gram. The vole was then transferred into a plastic tube of 15 cm diameter and transported individually to the adjacent enclosures where it was placed in the low-risk area, between the entrance to the nest-box and the food patch. Each vole remained in its enclosure for five days. The animal was then retrieved using live traps (Ugglan Special Traps n. 2, Grahnab AB, Hillerstorp, Sweden) and weighed.

Food trays were replaced every morning around 08.30 am. The sand was sieved, and recovered food items were dried in a drying cabinet at 60 °C for six hours. The dried food was cleaned of remaining sand and debris, and weighed to the nearest centigram to determine GUDs.

Food patches were monitored with motion-sensor, infra-red video-cameras (1/4″ CMOS Night Vision Camera, Detec Secure, Detec Handels GmbH, Witzenhausen, Germany). Recording started when the camera sensors detected movement in the feeding tray and continued for as long as the movement lasted. Based on video-monitoring we ascertained that all animals found and explored both food trays within the first 12 h of the first experimental day. One observer (VM) inspected all the recorded video footage, selected the videos in which the vole was using the tray, and quantified the following variables from each selected video with the software BORIS^87^: number of visits to each food patch, visit duration (sec), and proportion of time spent searching for food (e.g. digging, exploring the food patch while looking at the sand), eating (retrieving, handling and consuming hazelnuts), and exercising vigilance (cessation of feeding with head up and inspection of the surroundings^88^). Behaviours “searching” and “eating” were later pooled and considered as “foraging”. The analysis started when the vole entered the feeding tray with the front paws and ended when the vole left the tray with the posterior paws.

### Statistical analyses

We used restricted maximum-likelihood linear mixed effects models (LMMs) with a Gaussian error distribution to evaluate the relationship between giving-up densities (GUDs), duration of visits to the food patches, proportion of time spent foraging and proportion of time spent exercising vigilance, respectively and cognitive style/personality, risk area (high-risk vs low-risk), sex, mean-centred experimental day, and time of day (day vs night – except for the GUDs), all specified as fixed effects. For the variable “number of visits” we used maximum-likelihood generalized linear mixed effects models (GLMMs) with Poisson-distributed errors. We tested the significance of explanatory variables by comparing nested models with and without the respective variable using a likelihood ratio test (LRT). In all models, individual identity was added as random factor, specified as random intercept. We initially took into account the dependence of the two food trays within each experimental day by adding an additional random factor specifying individual experimental days. Since this factor did not improve the models’ AIC, it was removed from the reported models.

Duration of visits was log-transformed, and GUDs were reversed to food consumption (amount of food provided – amount of food left), and then log-transformed, to meet the normality assumption. Based on existing literature (e.g.^56^) and on preliminary data analyses, we included in the initial models 3- and 2-way interactions among all the explanatory variables except sex, that was never found to interact with any other factor. Analyses were first performed on the whole datasets; a 3-way interaction between the explanatory variables cognitive style/personality, risk area and experimental day was found in each case, so we re-ran the analyses on subsets of data including only fast individuals or only slow individuals, respectively. In these models, we excluded interactions stepwise if they proved non-significant based on log-likelihood ratio tests^89–92^. We report and discuss further only the results from these final models. Results of the initial 3-way models are reported in Table S1 in the Supplementary Material. All data analyses were conducted with R, version 3.2.3^90^ using the R packages lme4^91^, version 1.1–12, and nlme^92^, version 3.1–131. Visual inspection of residual plots did not reveal any obvious deviations from homoscedasticity or normality. In order to confirm that fast animals kept responding to the differential risk, we compared all measures of patch use on the last day between high- and low-risk food patches using a Mann-Whitney-U test. To complement the analysis of interactive effects, we also ran the same models on subsets of data including only the high-risk or the low-risk area. These results are presented in the Supplementary Material (Table S2).

We compared the overall food consumption, as well as changes in body mass between fast and slow individuals with Mann-Whitney-U tests. Since the initial body mass differed (fast individuals mean body mass ± SD: 24.7 ± 3.9 g; slow individuals mean body mass ± SD: 23.0 ± 4.3 g) we expressed the changes in body mass as percentage of the initial individual body mass. The accepted significance level was α ≤ 0.05.

### Ethical note

All animal experimentation was approved by the “Landesamt für Umwelt, Gesundheit und Verbraucherschutz Brandenburg” (reference number: V3-2347-44-2011) and the “Landesamt für Natur, Umwelt und Verbraucherschutz Nordrhein-Westfalen” (reference number: 84-02.04.2016.A253). Experiments were performed in accordance with all applicable international, national, and/or institutional guidelines for the use of animals, including the ASAB/ABS guidelines for the Use of Animals in Research.

## Supplementary information

Supplementary material

Supplementary Dataset 1

## Data Availability

Data are available as supplementary material.
