# Supplementary material for: Individual variation in cognitive style reflects foraging and anti-predator strategies in a small mammal
Source: Sci Rep. 2019 Jul 12;9:10157. doi: 10.1038/s41598-019-46582-1 (PMC6626059; doi:10.1038/s41598-019-46582-1)
Supplement: Supplementary file 1 — Supplementary material [file 41598_2019_46582_MOESM1_ESM.pdf]

SUPPLEMENTARY MATERIAL

Individual variation in cognitive style reflects foraging and anti-predator strategies in a small mammal

Mazza V., Jacob J., Dammhahn M., Zaccaroni M., & Eccard J. A.

Appendix A

Assessment of cognitive style and personality

We initially assessed 86 voles (45 males and 41 females) for olfactory associative learning. The test consisted of two tasks: an initial learning task and a reversal learning task. The reward was the chance to return to the safety of the home cage. The test arena was a plastic Y-maze (40 cm long, 5 cm wide, walls 15 cm high) with bent arms, so that the end of the arm was not visible from where the animals were released. One of the openings at the end of the maze was closed with wire mesh; the other had a mesh flap of the same material allowing the vole to exit the maze and enter the home cage. Different odour cues were placed inside each arm of the maze about 5 cm from the intersection. The side of the open door and associated positive cue were alternated each time a vole entered the maze, to avoid arm bias effects. For reversal learning, we switched the positive and the negative cues, so that the previously rewarded odour now led to the blocked door. Both tasks were considered successfully solved when the vole chose the arm leading to the home cage in seven out of 10 consecutive trials. Learning and reversal learning were expressed as learning and reversal learning scores, the number of trials necessary to reach the criterion in the two tasks.

We also assessed among-individual differences in activity, exploration and boldness using two standardized laboratory tests[13]: the open field test and the novel object test. The open field test[63] measures the activity and exploration of a novel, empty, circular arena. The novel object test measures the animal's boldness and neophobia when an unknown object is placed in a familiar environment. Both tests were repeated 15 days later and repeatability of the measured traits was assessed (boldness: $R = 0.67$, CI = 0.68-0.89, $p < 0.001$; activity: $R = 0.74$, CI = 0.65-0.84, $p < 0.001$). Boldness and activity correlated at the phenotypic level (mean trait value per individual: $r_S = 0.43$, $P < 0.001$).

Learning speed, flexibility and personality were closely related[36], with faster, inflexible learners being bolder and more active than slower, flexible learners. After completing these assessments, we used the learning and reversal learning scores and the personality to rank the animals. We selected those

with more pronounced traits for the study presented here (N = 30 males, N = 30 females). The groups differed in activity and boldness, as well as learning and reversal learning (Mann-Whitney-U test, all $p < 0.05$, figure S1 in the Supplementary Material).

Fig. S1 – Learning, reversal learning, activity and boldness scores of the two groups of bank voles. The mean (± SD) learning score for fast learners was 13.6 ± 1.8 and 20.1 ± 2.3 for slow learners. The mean reversal learning score was 19.5 ± 2.2 for fast learners and 13.9 ± 2.0 for slow learners. The mean boldness score was 0.83 ± 0.66 for fast learners and -0.63 ± 0.62 for slow learners. The mean activity score was 0.85 ± 0.75 for fast learners and -0.70 ± 0.72 for slow learners.

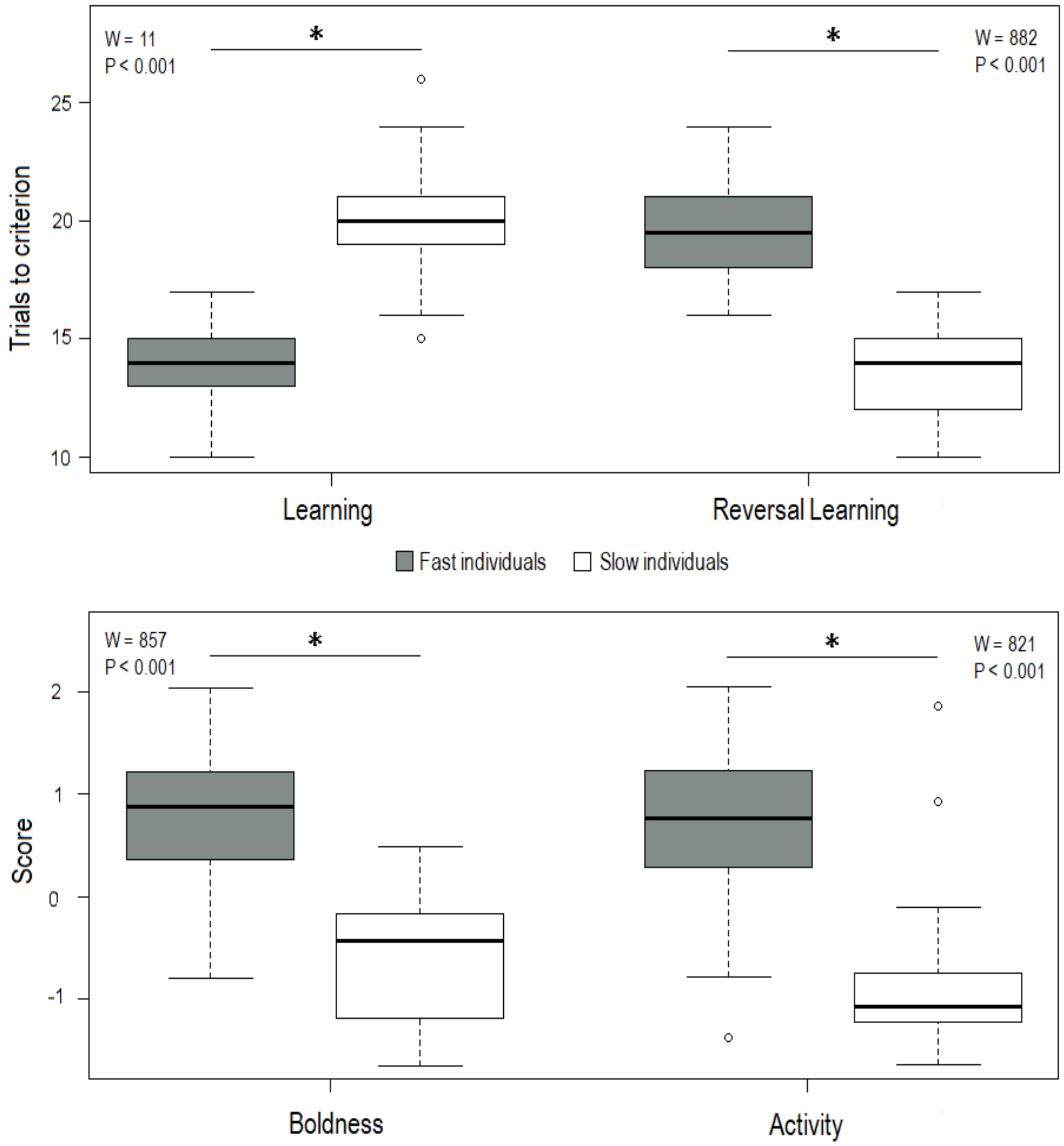

Table S1 – Results of the full models for giving-up densities (GUDs), number of visits, duration of visits, proportion of time spent foraging and in vigilance in relation to cognitive style/personality (fast vs slow), risk area (high-risk vs low-risk), experimental day, sex and time of day (day vs night) for 5 days of observations of 39 individual bank voles (*Myodes glareolus*) in outdoor enclosures. Statistically significant effects are highlighted in bold.

	**GUDs**					**Visit N**				**Visit Duration**				
	Estimate	SE	DF	F	P	Estimate	SE	z	P	Estimate	SE	DF	F	P
Intercept	0.64	0.03	1 345	1008.1	**< 0.001**	1.40	0.05	29.0	**< 0.001**	4.59	0.09	1 624	7137.24	**< 0.001**
Cognitive style/personality (Slow)	-0.01	0.03	1 36	20.4	**< 0.001**	-0.28	0.06	-5.0	**< 0.001**	0.52	0.11	1 36	1.40	0.244
Area (High-risk)	0.19	0.02	1 345	806.1	**< 0.001**	-0.90	0.08	-10.8	**< 0.001**	-0.77	0.06	1 624	670.65	**< 0.001**
Experimental day	-0.04	0.01	1 345	39.3	**< 0.001**	0.22	0.02	9.1	**< 0.001**	0.21	0.02	1 624	368.97	**< 0.001**
Sex (M)	0.01	0.03	1 36	0.1	0.75	-0.10	0.04	-2.4	**0.02**	-0.06	0.11	1 36	0.17	0.686
Time of day (Night)			/			0.07	0.05	1.3	0.21	0.08	0.04	1 624	100.03	**< 0.001**
Cognitive style/personality:Area	0.29	0.02	1 345	162.2	**< 0.001**	-0.82	0.10	-8.2	**< 0.001**	-1.37	0.07	1 624	286.04	**< 0.001**
Cognitive style/personality:Experimental day	-0.03	0.01	1 345	43.0	**< 0.001**	-0.01	0.04	-0.3	0.78	0.15	0.03	1 624	1.02	0.314
Area:Experimental day	-0.02	0.01	1 345	53.4	**< 0.001**	0.01	0.04	0.3	0.74	-0.02	0.03	1 624	54.76	**< 0.001**
Area:Time of day			/			1.10	0.09	11.9	**< 0.001**	0.67	0.07	1 624	95.88	**< 0.001**
Cognitive style/personality:Area:Experimental day	0.16	0.02	1 345	99.6	**< 0.001**	-0.49	0.07	-7.1	**< 0.001**	-0.45	0.05	1 624	74.78	**< 0.001**

	**Foraging**					**Vigilance**				
	Estimate	SE	DF	F	P	Estimate	SE	DF	F	P
Intercept	73.38	1.48	1 625	4860.1	**< 0.001**	26.62	1.48	1 625	3249.2	**< 0.001**
Cognitive style/personality (Slow)	-26.90	1.76	1 36	138.9	**< 0.001**	26.90	1.76	1 36	138.9	**< 0.001**
Area (High-risk)	-25.68	1.19	1 625	433.8	**< 0.001**	25.68	1.19	1 625	433.8	**< 0.001**
Experimental day	4.79	0.57	1 625	52.7	**< 0.001**	-4.79	0.57	1 625	52.7	**< 0.001**
Sex (Male)	0.75	1.58	1 36	0.2	0.67	-0.75	1.58	1 36	0.2	0.67
Time of day (Night)	2.61	0.91	1 625	10.1	**0.002**	-2.61	0.91	1 625	10.1	**0.002**
Cognitive style/personality:Area	13.15	1.95	1 625	76.6	**< 0.001**	-13.15	1.95	1 625	76.6	**< 0.001**
Cognitive style/personality:Experimental day	-3.72	0.84	1 625	66.0	**< 0.001**	3.72	0.84	1 625	66.0	**< 0.001**
Area:Experimental day	0.06	0.84	1 625	8.3	**0.004**	-0.06	0.84	1 625	8.3	**0.004**
Cognitive style/personality:Area:Experimental day	-5.58	1.41	1 625	15.7	**< 0.001**	5.58	1.41	1 625	15.7	**< 0.001**

Table S2 – Giving-up densities (GUDs), number of visits, duration of visits, proportion of time spent foraging and in vigilance in relation to cognitive style/personality (fast vs slow), experimental day, sex and time of day (day vs night) in the high-risk and low-risk area for 5 days of observations of 39 individual bank voles (*Myodes glareolus*) in outdoor enclosures. Statistically significant effects are highlighted in bold.

	Low risk						High risk					
**GUDs**	Estimate	SE	DF		F	P	Estimate	SE	DF		F	P
Intercept	0.65	0.03	1	154	1213.9	**< 0.001**	0.45	0.03	1	154	341.0	**< 0.001**
Cognitive style/personality (Slow)	-0.01	0.04	1	36	0.0	0.88	0.28	0.04	1	36	64.2	**< 0.001**
Experimental day	-0.04	0.01	1	154	118.9	**< 0.001**	-0.06	0.01	1	154	0.7	0.41
Sex (Male)	0.02	0.04	1	36	0.3	0.57	-0.001	0.04	1	36	0.0	0.97
Cognitive style/personality:Experimental day	-0.03	0.01	1	154	7.5	**0.01**	0.13	0.01	1	154	175.8	**< 0.001**
**Visit N**	Estimate	SE			z	P	Estimate	SE			z	P
Intercept	1.40	0.05			27.9	**< 0.001**	0.50	0.08			6.1	**< 0.001**
Cognitive style/personality (Slow)	-0.29	0.05			-5.4	**< 0.001**	-1.10	0.09			-12.0	**< 0.001**
Experimental day	0.22	0.02			11.4	**< 0.001**	0.24	0.03			8.7	**< 0.001**
Sex (Male)	-0.10	0.05			-1.8	0.07	-0.11	0.08			-1.5	0.14
Time of day (Night)	0.07	0.05			1.3	0.21	1.17	0.08			15.3	**< 0.001**
Cognitive style/personality:Experimental day			/				-0.50	0.06			-8.8	**< 0.001**
**Visit Duration**	Estimate	SE	DF		F	P	Estimate	SE	DF		F	P
Intercept	4.59	0.13	1	346	4243.2	**< 0.001**	3.81	0.09	1	241	5255.8	**< 0.001**
Cognitive style/personality (Slow)	0.52	0.15	1	36	12.4	**0.001**	-0.89	0.11	1	36	48.2	**< 0.001**
Experimental day	0.21	0.02	1	346	473.6	**< 0.001**	0.21	0.02	1	241	43.8	**< 0.001**
Sex (Male)	-0.07	0.15	1	36	0.2	0.65	-0.05	0.11	1	36	0.0	0.83
Time of day (Night)	0.08	0.04	1	346	5.2	**0.02**	0.77	0.04	1	241	322.7	**< 0.001**
Cognitive style/personality:Experimental day	0.15	0.03	1	346	34.3	**< 0.001**	-0.32	0.03	1	241	85.4	**< 0.001**
**Foraging**	Estimate	SE	DF		F	P	Estimate	SE	DF		F	P
Intercept	73.96	1.64	1	346	4516.0	**< 0.001**	46.63	1.83	1	241	2020.6	**< 0.001**
Cognitive style/personality (Slow)	-26.91	1.87	1	36	206.6	**< 0.001**	-13.71	2.15	1	36	30.9	**< 0.001**
Experimental day	4.79	0.51	1	346	68.6	**< 0.001**	4.92	0.69	1	241	12.9	**< 0.001**
Sex (Male)	0.30	1.87	1	36	0.0	0.88	1.64	2.03	1	36	0.8	0.39
Time of day (Night)	1.87	1.05	1	346	3.2	0.08	3.78	1.59	1	241	4.7	**0.03**
Cognitive style/personality:Experimental day	-3.73	0.75	1	346	24.8	**< 0.001**	-9.21	1.26	1	241	53.2	**< 0.001**
**Vigilance**	Estimate	SE	DF		F	P	Estimate	SE	DF		F	P
Intercept	26.04	1.64	1	346	1604.0	**< 0.001**	53.37	1.83	1	241	2939.0	**< 0.001**
Cognitive style/personality (Slow)	26.91	1.87	1	36	206.6	**< 0.001**	13.71	2.15	1	36	30.9	**< 0.001**
Experimental day	-4.79	0.51	1	346	68.7	**< 0.001**	-4.92	0.69	1	241	12.9	**< 0.001**
Sex (Male)	-0.30	1.87	1	36	0.0	0.88	-1.64	2.03	1	36	0.8	0.39
Time of day (Night)	-1.87	1.05	1	346	3.2	0.08	-3.78	1.59	1	241	4.7	**0.03**
Cognitive style/personality:Experimental day	3.73	0.75	1	346	24.8	**< 0.001**	9.21	1.26	1	241	53.2	**< 0.001**
